# Supplementary material for: Structural Basis for Amyloid Fibril Assembly by the Master Cell-Signaling Regulator Receptor-Interacting Protein Kinase 1
Source: Nat Commun. Author manuscript; Available in PMC 2025 Nov 14. (PMC12575786; doi:10.1038/s41467-025-64621-6)

Supporting Information for:

Structural Basis for Amyloid Fibril Assembly by the Master Cell-Signaling Regulator Receptor-Interacting Protein Kinase 1

Paula Polonio[1,2], Jorge Pedro López-Alonso[3,4], Hanxing Jiang[3], Sara Andrés-Campos[1], Fátima C. Escobedo-González[1], Gustavo A. Titaux-Delgado[1], Iban Ubarretxena-Belandia[3,5*], Miguel Mompeán[1*]

*Correspondence: mmompean@iqf.csic.es and ivan.ubarrechena@ehu.eus

1. Instituto de Química Física Blas-Cabrera (IQF-CSIC), Madrid, Spain
2. Universidad Autónoma de Madrid, Escuela de Doctorado, Madrid, Spain
3. Instituto Biofisika (UPV/EHU, CSIC), Leioa, Spain
4. Basque Resource for Electron Microscopy, Leioa, Spain
5. Ikerbasque Foundation for Science, Bilbao, Spain

Contents:

Supplementary Figure 1 | SSNMR structure calculation.

Supplementary Figure 2 | Workflow of cryo-EM data processing and helical reconstruction.

Supplementary Table 1 | SSNMR acquisition parameters**.**

Supplementary Table 2 | Restraints identified in the spectra.

Supplementary Table 3 | SSNMR structure calculation statistics.

Supplementary Table 4 | Cryo-EM data collection, refinement and validation statistics.

Supplementary Note 1 | Glossary of solid-state NMR acronyms and assignment strategy.

Figure S1 | SSNMR structure calculation. a 2D ^{13}C-^{13}C CORD spectra of hRIPK1 showing specific unambiguous (black), ambiguous (blue), and intraresidue cross peaks (purple) at 100 ms (cyan) and 500 ms (grey) mixing time. **b** Preliminary structure calculated using 36 torsion angle restraints and 7 unambiguous distance restraints (black dashed lines). Blue dashed lines represent low-ambiguity restraints that can be mapped onto the structure. **c** Full structure calculation with automatic assignment of all ambiguous distance restraints along with the restraints shown in panel b, and using this preliminary model as a starting structure or "seed". Source data are provided as a Source Data file.

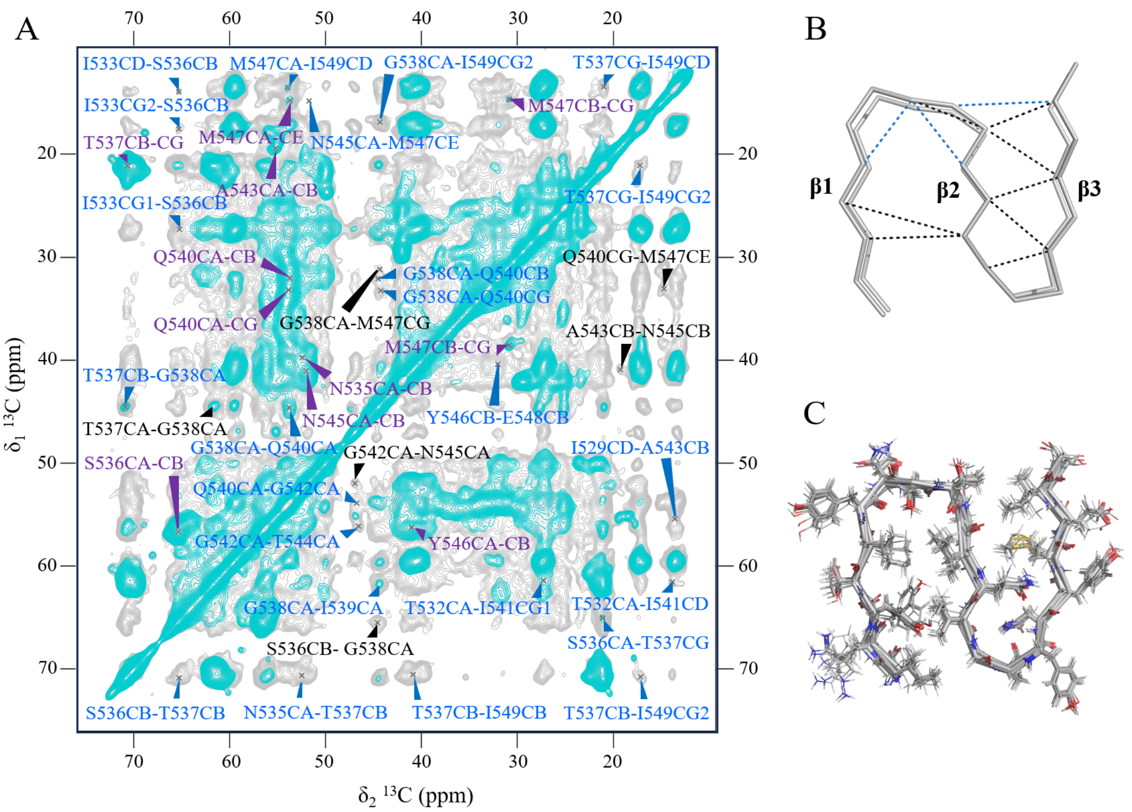

Figure S2 | Stereo view of the hRIPK1(532–561) core structure ensemble

Stereo image (cross-eyed view) showing the superposition of the 20 lowest-energy structures calculated by solid-state NMR for the hRIPK1 fibril protomer (residues 529–550). Backbone atoms are shown in stick representations in gray, with side chains in stick representation (H, white; C, gray; N, blue; O, red; S, yellow). The RHIM tetrad IQIG (residues 539-542) is shown in cartoon representation in wheat.

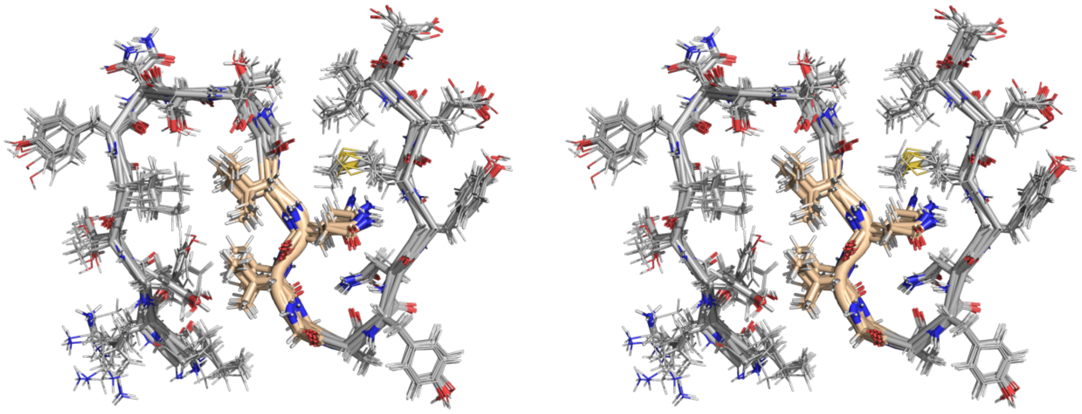

Figure S3 | Workflow of cryo-EM data processing and helical reconstruction. **a** A total of 2,814,672 filament segments were automatically picked from 8,650 micrographs and subjected to iterative 2D classification to remove noise and poor-quality segments. An initial 3D model was generated using ab initio reconstruction without imposing symmetry constraints. Helical parameters (rise and twist) were determined through a symmetry search, followed by refinement using non-uniform reconstruction. To improve the map, several rounds of local and global CTF (contrast transfer function) refinement, as well as reference-based motion correction, were performed. The local resolution of the final map, estimated using the 0.5 FSC (Fourier Shell Correlation) criterion, is shown. **b** 3D classes obtained from the main reconstruction. No evidence of structural polymorphism was found, as all classes exhibited the same helical symmetry, in agreement with the observation of a unique single set of resonance in the SSNMR spectra. Minor differences observed between classes likely reflect artifacts from local fibril curvature affecting the reconstruction near the edges of the extracted segments.

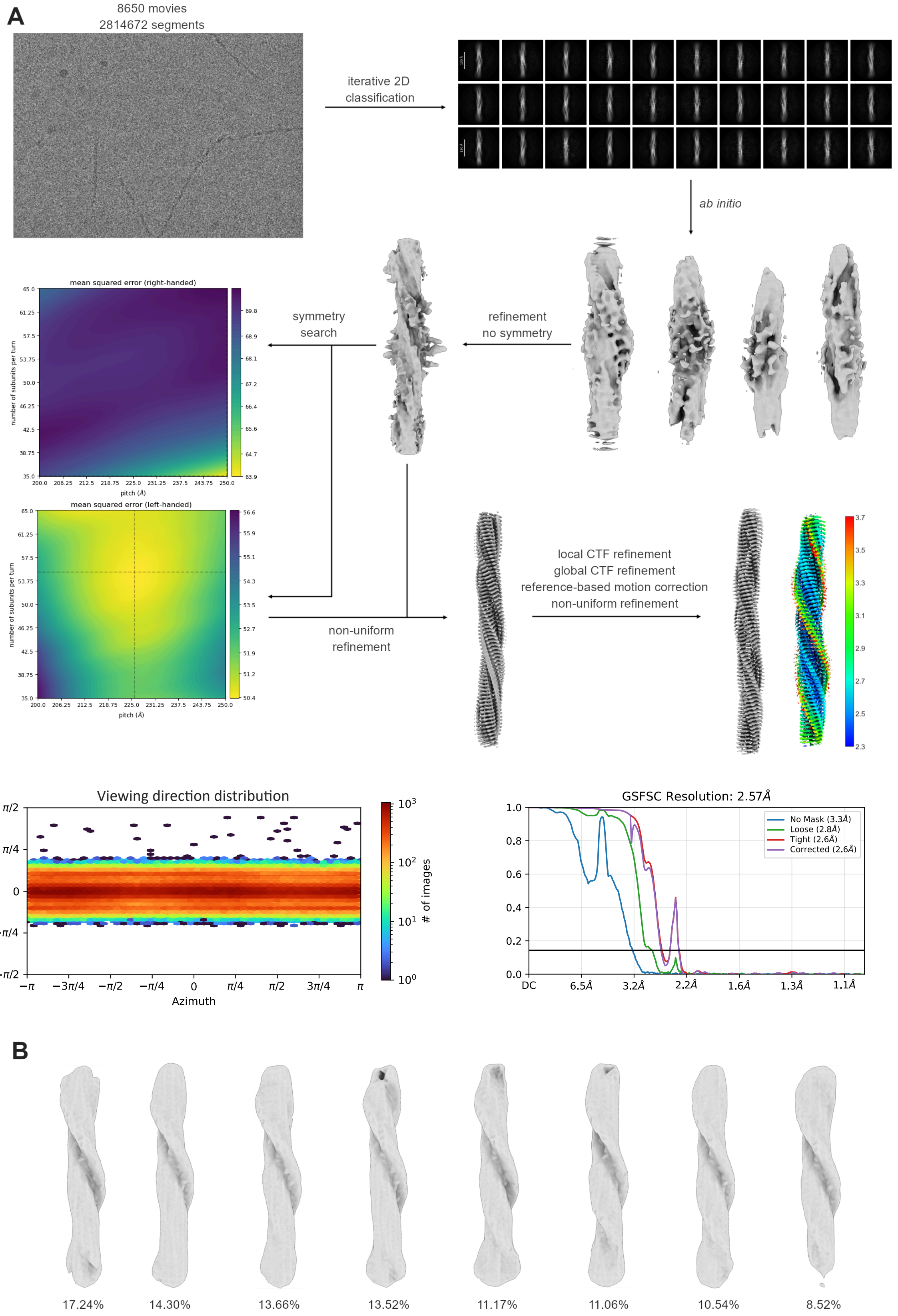
A
8650 movies
2814672 segments
iterative 2D
classification
ab initio
refinement
no symmetry
symmetry
search
mean squared error (right-handed)
mean squared error (left-handed)
number of subunits per turn
pitch (Å)
non-uniform
refinement
local CTF refinement
global CTF refinement
reference-based motion correction
non-uniform refinement
Viewing direction distribution
Azimuth
# of images
GSFSC Resolution: 2.57Å
No Mask (3.3Å)
Loose (2.8Å)
Tight (2.6Å)
Corrected (2.6Å)
DC
6.5Å
3.2Å
2.2Å
1.6Å
1.3Å
1.1Å
B
17.24%
14.30%
13.66%
13.52%
11.17%
11.06%
10.54%
8.52%

Table S1 | SSNMR acquisition parameters.

14.1 T (600 MHz 1H frequency), 3.2 mm HCN CPMAS CryoProbe										
Experiment	NS	MAS (kHz)	d1 (s)	Acq time (ms)	Sweep width (kHz)	Dec power (kHz)	Mixing (ms)	CP time (ms)	Field Strength (kHz)	Sample T (K)
2D NCA	8	14	2	t2: 15; t1: 11	ω2: 52.6; ω1: 2.8	100	-	HN: 1.00; NC: 1.50	H: 54; N: 40 (transfer 1); N: 4; CA: 18 (transfer 2)	315
2D NCACX	8	14	2	t3: 15.0; t1: 7.9	ω3: 52.6; ω2: 3.5; ω1: 2.8	100	50	HN: 1.00; NC: 1.50	H: 54; N: 40 (transfer 1); N: 4; CA: 18 (transfer 2)	315
3D NCACX	8	14	2	t3: 15.0; t2: 8.0; t1: 7.9	ω3: 52.6; ω2: 3.5; ω1: 2.8	100	50	HN: 1.00; NC: 1.50	H: 54; N: 40 (transfer 1); N: 4; CA: 18 (transfer 2)	315
3D NCOCX	8	14	2	t3: 15.0; t2: 8.0; t1: 7.9	ω3: 52.6; ω2: 3.5; ω1: 2.8	100	50	HN: 1.00; NC: 1.50	H: 54; N: 40 (transfer 1); N: 8.4; CA: 5.6 (transfer 2)	315
3D CANCOCX	64	14	2	t3: 17.4; t2: 7.9; t1: 6.0	ω3: 58.8; ω2: 2.8; ω1: 4.7	100	50	HC: 1.20; CN: 1.25; NC: 7.00	H: 69; CA: 55 (transfer 1); CA: 3; N: 11 (transfer 2); N: 8.4; CO: 5.6 (transfer 3)	315
2D CORD	4	14	2	t2: 20; t1: 17	ω2: 44.2; ω1: 35	100	5, 20, 100, 500	HC: 1.75	H: 69 ; C: 55	315

Table S2 | Restraints identified in the spectra.

Unambiguous restraints

Residue i	Residue i type	Atom i type	Residue j	Residue j type	Atom j type	Distance restraints	
532	THR	C	541	ILE	CD	long-range	*
536	SER	CA	538	GLY	CA	medium-range	*
536	SER	CB	538	GLY	CA	medium-range	*
538	GLY	CA	550	GLY	CA	long-range	*
538	GLY	CA	547	MET	CG	long-range	*
538	GLY	CA	547	MET	CE	long-range	*
538	GLY	C	547	MET	CE	long-range	*
540	GLN	CG	545	ASN	CB	long-range	*
540	GLN	CG	547	MET	CE	long-range	*
540	GLN	CD	547	MET	CE	long-range	*
542	GLY	CA	545	ASN	CA	medium-range	*
543	ALA	CB	545	ASN	CB	medium-range	

Ambiguous restraints

Residue i	Residue i type	Atom i type	Residue j	Residue j type	Atom j type	Distance restraints	
533	ILE	CA	536	SER	CB	medium-range	
533	ILE	CB	536	SER	CB	medium-range	
533	ILE	CG1	536	SER	CB	medium-range	**
533	ILE	CG2	536	SER	CB	medium-range	**
533	ILE	CD	536	SER	CB	medium-range	**
533	ILE	CD	536	SER	CA	medium-range	**
535	ASN	CA	537	THR	CB	medium-range	
536	SER	CA	539	ILE	CG1	medium-range	**
536	SER	CB	539	ILE	CA	medium-range	
536	SER	CB	539	ILE	CB	medium-range	
536	SER	CB	539	ILE	CG1	medium-range	**
536	SER	CB	539	ILE	CG2	medium-range	**
537	THR	CA	535	ASN	CA	medium-range	
537	THR	CB	535	ASN	CA	medium-range	
537	THR	CA	539	ILE	CG1	medium-range	
537	THR	CB	539	ILE	CG1	medium-range	
537	THR	CA	539	ILE	CD	medium-range	
537	THR	CB	539	ILE	CD	medium-range	
537	THR	CG	539	ILE	CD	medium-range	
537	THR	CB	550	GLY	CA	long-range	
537	THR	CG	550	GLY	CA	long-range	**
537	THR	CB	549	ILE	CB	long-range	
537	THR	CB	549	ILE	CG2	long-range	
537	THR	CG	549	ILE	CG2	long-range	
537	THR	CG	549	ILE	CD	long-range	
538	GLY	CA	540	GLN	CA	medium-range	
538	GLY	CA	540	GLN	CB	medium-range	
538	GLY	CA	540	GLN	CG	medium-range	
538	GLY	CA	547	MET	CA	long-range	
538	GLY	CA	547	MET	CB	long-range	
538	GLY	CA	549	ILE	CG2	long-range	
540	GLN	CA	542	GLY	CA	medium-range	
540	GLN	CA	547	MET	CE	long-range	**
540	GLN	CB	547	MET	CE	long-range	**
542	GLY	CA	544	TYR	CA	medium-range	
542	GLY	CA	545	ASN	CB	medium-range	**
545	ASN	CA	547	MET	CE	medium-range	
546	TYR	CB	548	GLU	CB	medium-range	
547	MET	CA	549	ILE	CD	medium-range	

Medium-range restraints (|i-j| > 1 and |i-j| < 5): pairs separated by 2-4 residues in the sequence
Long-range restraints (|i-j| ≥ 5): pairs that are at least 5 residues apart in the sequence
* Distance restraints used in preliminary structure calculation
** Low ambiguity restraints that were resolved based on the preliminary model

Table S3 | SSNMR structure calculation statistics.

	**hRIPK1 fibrils**
**NMR distance and dihedral constraints**	
Distance constraints	121
Intra-residue	4
Inter-residue	117
Sequential ($\|i-j\| = 1$)	44
Medium-range ($\|i-j\| < 4$)	48
Long-range ($\|i-j\| > 5$)	25
Intermolecular	0
Hydrogen bonds	0
Total dihedral angle restraints	35
ϕ	17
ψ	18
**Structure statistics**	
Violations (mean and s.d.)	
Distance constraints (Å)	0.016 ± 0.02
Dihedral angle constraints (º)	0.34 ± 1.03
Max. dihedral angle violation (º)	5.40
Max. distance constraint violation (Å)	0.40
Deviations from idealized geometry	
Bond lengths (Å)	0.008 ± 0.001
Bond angles (º)	1.0 ± 0.1
Impropers (º)	0.4 ± 0.2
Average pairwise r.m.s. deviation** (Å)	
Heavy	0.59
Backbone	0.33

**Pairwise r.m.s. deviation was calculated among 20 refined structures.

Table S4 | Cryo-EM data collection, refinement and validation statistics.

	Human RIPK1<br>(EMDB-52356)<br>(PDB 9HR6)
**Data collection and processing**	
Magnification	165000
Voltage (kV)	300
Electron exposure (e–/Å^2)	49.3
Defocus range (μm)	0.8-1.6
Pixel size (Å)	0.5054
Axial symmetry	C1
Twist (degree)	-7,319
Rise (Å)	4,667
Initial segment images (no.)	2814672
Final segment images (no.)	460173
Map resolution (Å)	2.57
FSC threshold	0.143
Map resolution range (Å)	2.32-2.86
**Refinement**	
Initial model used	*In silico* (ModelAngelo)
Model resolution (Å)	2.57
FSC threshold	0.143
Model resolution range (Å)	2.32-2.86
Map sharpening *B* factor (Å^2)	-57.4
Model composition	
Non-hydrogen atoms	930
Protein residues	120
Ligands	0
B factor (Å^2)	65.70
R.M.S. deviations	
Bond lengths (Å)	0.004
Bond angles (°)	0.789
Validation	
MolProbity score	0.82
Clashscore	0
Poor rotamers (%)	0
Ramachandran plot	
Favored (%)	95.45
Allowed (%)	4.55
Disallowed (%)	0.00

Note S1 | Glossary of solid-state NMR acronyms and assignment strategy

SSNMR: Solid-State Nuclear Magnetic Resonance.

CPMAS: Cross-Polarization Magic-Angle Spinning.

NCACX: Correlates nitrogen (N) to Cα and other carbons (C).

NCOCX: Correlates nitrogen (N) to carbonyl (C′) and other carbons.

CANCOCX: Detects Cα–N–C′–C connectivities.

The NCACX experiment yielded correlations within each residue, capturing N_i, CA_i, and CX_i signals for the residue i, where CX_i represents both CO_i and $^{13}C_i$ side-chain atoms. The NCOCX experiment linked the ^{13}C atoms of a residue i to the nitrogen atom of the next residue (N_{i+1}), while the CANCOCX experiment bridged CA_{i+1} and N_{i+1} with the ^{13}C atoms of residue i, enabling sequential connectivity throughout the fibril core. The schematic representation of the magnetization transfer in these three 3D experiments used for backbone sequential assignment is shown below:

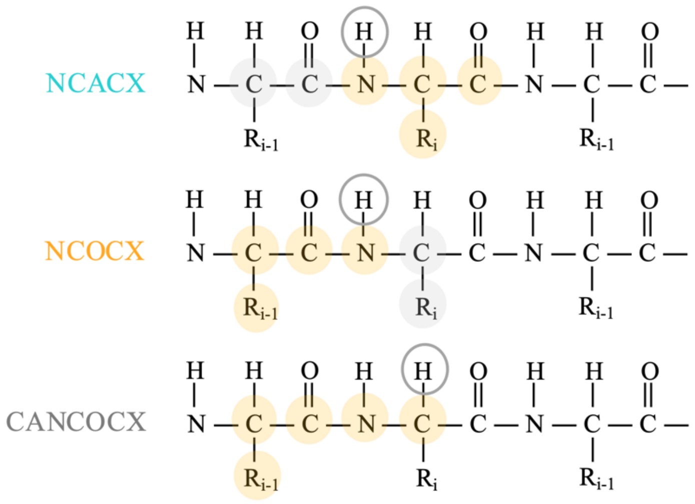

Supplement: Supporting Information [file EMS209321-supplement-Supporting_Information.pdf]
